# The Effects of Exoskeleton Assisted Knee Extension on Lower-Extremity Gait Kinematics, Kinetics, and Muscle Activity in Children with Cerebral Palsy

**DOI:** 10.1038/s41598-017-13554-2

**Published:** 2017-10-18

**Authors:** Zachary F. Lerner, Diane L. Damiano, Thomas C. Bulea

**Affiliations:** 1 Functional and Applied Biomechanics Section, Rehabilitation Medicine Department, National Institutes of Health, Bethesda, MD USA; 2 Department of Mechanical Engineering, Northern Arizona University, Flagstaff, AZ USA

## Abstract

Individuals with cerebral palsy often exhibit crouch gait, a debilitating and inefficient walking pattern marked by excessive knee flexion that worsens with age. To address the need for improved treatment, we sought to evaluate if providing external knee extension assistance could reduce the excessive burden placed on the knee extensor muscles as measured by knee moments. We evaluated a novel pediatric exoskeleton designed to provide appropriately-timed extensor torque to the knee joint during walking in a multi-week exploratory clinical study. Seven individuals (5–19 years) with mild-moderate crouch gait from cerebral palsy (GMFCS I-II) completed the study. For six participants, powered knee extension assistance favorably reduced the excessive stance-phase knee extensor moment present during crouch gait by a mean of 35% in early stance and 76% in late stance. Peak stance-phase knee and hip extension increased by 12° and 8°, respectively. Knee extensor muscle activity decreased slightly during exoskeleton-assisted walking compared to baseline, while knee flexor activity was elevated in some participants. These findings support the use of wearable exoskeletons for the management of crouch gait and provide insights into their future implementation.

## Introduction

Many individuals with cerebral palsy (CP), the most prevalent cause of physical disability in childhood^1^, present an increasingly flexed or “crouched” gait pattern throughout the lifespan^2^. This pathological gait pattern, known as crouch gait, increases the metabolic cost of transport^3^ and is associated with knee pain and degradation due to elevated joint loads^4^. Despite efforts to manage crouch gait surgically^5,6^ and with physical therapy^7^, walking deficits generally remain after treatment such that approximately 50% of affected individuals lose the ability to walk independently in adulthood^8^. Effective gait rehabilitation remains a major clinical challenge in CP^9^.

Robotic exoskeletons offer the ability to augment human locomotion by applying appropriately timed and scaled torques to lower-extremity joints^10–12^. Exoskeletons have been designed for healthy individuals, aiming to improve walking economy^13–15^ and assist in load carriage^16–18^, and for individuals with disabilities, seeking to aid mobility and improve rehabilitation^19,20^. While exoskeleton-aided gait rehabilitation research has primarily focused on adults after stroke and spinal cord injury, powered exoskeletons may offer a unique alternative to existing treatments of pediatric gait disorders caused by CP.

Research utilizing gait analysis and musculoskeletal modeling suggests that the flexed posture during crouch gait reduces the capacity of the hip and knee extensor muscles to extend the lower-extremity joints during stance^21^. Crouch gait is also associated with elevated knee extensor muscle forces and increased knee extensor moments^22^, diminished contribution from the ankle plantar flexors, and the reliance of more proximally located muscles for forward progression^23,24^. In theory, these findings suggest that treating crouch via robotic knee extension assistance may reduce the excessive burden placed on the knee extensor muscles, improve the ability of several lower-extremity muscles to transfer mechanical energy from the legs to the center of mass, make walking more efficient, and prolong walking ability^25^. A robotic treatment strategy is different from the existing approaches to managing crouch gait, such as orthopaedic surgery, which alters muscle action via soft-tissue modification^26^ or boney realignment^5^, physical therapy, which seeks to strengthen muscles and improve coordination^27^, and passive bracing, which restricts motion^28^. Gait analysis has shown that multi-level surgery^26^ and orthotics^28^ can improve knee extension and reduce knee extensor moments in the short term, yet long term improvements are more difficult to maintain^29^. By reducing requirements for body-weight support and stability maintenance from the over-burdened extensor muscles, powered assistance may provide the opportunity to train coordinated and selective muscle control in natural environments and for longer sustained periods. However, complexity of the neuromuscular system, including the actions of bi-articular muscles, combined with impaired motor control and altered muscle physiology in individuals with CP^30^ make it difficult to predict how robotic assistance at the knee may affect the biomechanical behavior of the entire lower limb within and across individuals.

The purpose of our study was to evaluate how extension assistance from a powered knee exoskeleton affects lower limb joint dynamics in children and adolescents with crouch gait from CP. Our primary hypothesis was that providing additional extensor torque to the knee joint during the stance phase of crouch gait would result in improved knee extension and reduced knee extensor moments. To evaluate this hypothesis, we implemented a novel pediatric exoskeleton in a 6-visit exploratory clinical study, collected motion capture, force plate, and electromyography measurements, and completed an inverse dynamics analysis.

## Methods

### Study and Participant Information

Approval for this study was granted by the National Institutes of Health (NIH) Institutional Review Board under protocol #13-CC-0210. All research was carried out in accordance with the guidelines and regulations of the NIH. We recruited children and adolescents with a diagnosis of crouch gait from spastic diplegic CP. Seven participants met our inclusion criteria and completed our exploratory clinical study, which included the Gross Motor Function Classification System (GMFCS) level of I or II and the absence of any health condition, besides CP, that could impact safety. We obtained informed assent from each participant and consent from their parents. Participant information is listed in Table 1.

**Table 1:** Participant information.

Patient (ID)	Age (yrs)	Gender	Height (m)	Body Mass (kg)	GMFCS^a^ Level	Baseline Condition	MAS^b^ (Spasticity)	Strength (Nm/kg)		Knee ROM^c^ (°)
								*Extensor*	*Flexor*	
P1	6	M	1.11	20.0	II	AFO	0/0	0.68	0.31	69
P2	11	F	1.56	40.8	II	Shod	1+/1+	1.11	0.29	114
P3	19	F	1.48	65.1	II	Shod	1/1	0.29	0.05	88
P4	5	M	1.15	20.6	II	AFO	1+/1	0.69	0.32	90
P5	12	M	1.72	69.3	I	Shod	1/1	0.94	0.55	113
P6	11	M	1.35	32.0	II	Shod	2/1+	1.08	0.39	93
P7	10	F	1.48	42.5	II	AFO	1+/1+	0.38	0.27	102

^a^GMFCS: Gross Motor Function Classification Scale. AFO: ankle-foot orthosis was worn under shoes. ^b^MAS: Modified Ashworth Scale (clinical measure of spasticity) for knee flexors of the more/less affected limbs. Muscle strength, measured at the mid-point of range of motion, for knee extensor/flexor muscles are averaged across limbs. ^c^ROM: Range of Motion for the knee joint, averaged across limbs.

**Figure 1:**
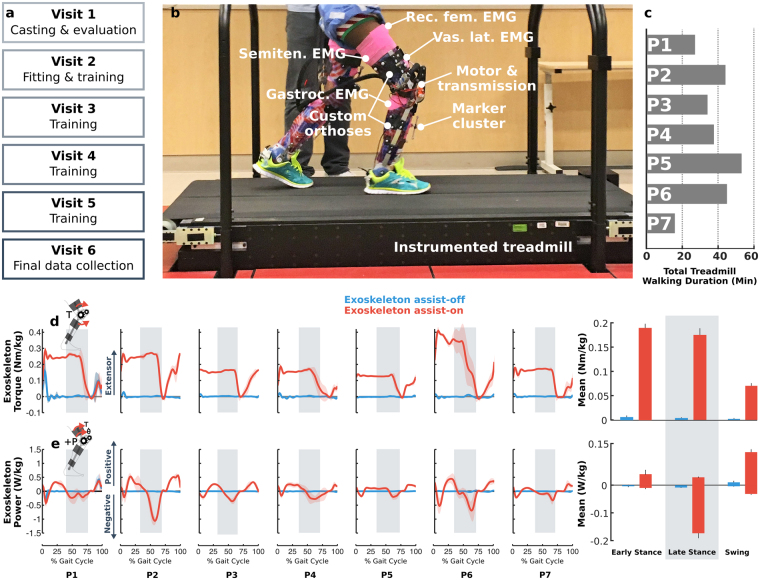
(**a**) Visits completed by study participants. Experimental data presented in this study are from visit 6.(**b**) The experimental setup. Participants walked on an instrumented treadmill with the exoskeleton tethered to a power supply while kinetic, kinematic, and electromyographic data were recorded. (**c**) Combined treadmill walking durations across study visits for each participant. Experimentally measured exoskeleton torque (**d**) and power (**e**) across the knee joint during walking with exoskeleton assist-on (red) and assist-off (blue) plotted vs. percent gait cycle for each individual’s more affected limb. Line shading indicates ±1 standard deviation. Vertical gray shading indicates late stance phase. Bar plots on the right depict the bilateral group-level summary (for all limbs) averaged across gait phases. Error bars indicate standard error. A small amount of non-zero toque was transiently present immediately before and after foot contact for P1 due to a controller initialization error.

Participants completed six visits at the NIH Clinical Center over the course of 8–12 weeks (Fig. 1a). The first visit included a clinical evaluation and lower-extremity casting for fabrication of custom thermoplastic braces. For subsequent training visits, each lasting 2–3 hours, the participants practiced walking with the exoskeleton over-ground and on a treadmill (Fig. 1b) with and without assistance as tolerated. Seated breaks, lasting approximately 5–15 minutes, were provided as frequently as needed to minimize fatigue. Actual walking duration for each participant is presented in Fig. 1c. The experimental biomechanics data presented in this study were collected on the sixth visit during treadmill walking. Treadmill speed and level of exoskeleton extension torque were established based on participant preference over the course of the training visits.

### Robotic Exoskeleton

We previously reported on the development of this novel powered lower-extremity exoskeleton intended to treat crouch gait by providing extension torque at the knee joint^31^ and on its ability to increase knee extension during over-ground walking in children with CP^32^. The exoskeleton contains a motor with planetary gear head connected to a custom chain-and-sprocket transmission assembly that is mounted at the knee to custom-molded thermoplastic braces (Fig. 1b). The exoskeleton’s ankle joint is a simple hinge set for free rotation. A finite state machine differentiates between the stance, early swing (knee flexion), and late swing (knee extension) phases of gait using data from an embedded force sensitive resistor and knee angle encoder. A proportional-integral-derivative control algorithm regulates the torque output during each state using the experimentally measured torque from a reaction torque sensor mounted at the knee joint. Participants with AFOs did not use them with the exoskeleton. The ankle joint included in the exoskeleton was set to free rotation for all participants to isolate the effects of knee extension assistance for this study. Individuals used their own athletic shoes at the baseline condition, while athletic shoes for use with the exoskeleton were supplied.

We tested several exoskeleton conditions during our protocol including assistance provided during stance only, late-swing only, and combined stance & late-swing. For the present study, we only report the analysis of the stance & late swing assist condition because it resulted in the greatest overall improvement in knee angle during over-ground walking^32^ (henceforth referred to as “exoskeleton assist-on”). In this mode, a constant extensor torque was specified for each participant during stance, to support the body and extend the knee during mid-stance, and late-swing, to extend the knee prior to initial foot contact (Fig. 1d). The magnitude of each participant’s assistive torque was adjusted after each of the first several walking trials, the number of which varied by participant, based on the objective of maximizing knee extension while also maintaining walking stability.

During the exoskeleton assist-off condition and in the early swing portion of the gait cycle during exoskeleton assist-on, we specified a zero-torque set-point, which compensated for device inertia and friction, resulting in resistance-free articulation to allow for natural knee flexion in early swing^31^. The device remained tethered to a power supply for this study to ensure uninterrupted function.

### Experimental Biomechanics Data

Gait analysis included motion capture, force plate, and electromyography measurement during treadmill walking. For kinematic analysis, reflective markers were placed on the lower-extremity, pelvis and torso as in ref.^33^; specifically on the medial and lateral aspects of the ankle and knee joints, 3 on the foot, 4 on the pelvis, and 3 on the torso. Clusters comprised of 4 non-collinear markers were placed on each shank and thigh. Marker trajectories were recorded at 100 Hz from 10 motion capture cameras (Vicon Motion Systems, Oxford, UK). Muscle activity was collected bilaterally from the medial gastrocnemius, vastus lateralis, semitendinosus, and rectus femoris using a wireless EMG system (Trigno, Delsys, Boston, MA) recording at 1000 Hz. Kinetic data were collected at 1000 Hz from an instrumented split-belt treadmill (Bertec, Columbus, OH). Kinematic, kinetic, EMG, and exoskeleton data were synchronized in the Vicon system.

### Data Processing

Marker data were low-pass filtered at 6 Hz, while force plate data were low-pass filtered at 12 Hz. EMG data were band-pass filtered at 15–380 Hz, full-wave rectified, and low-pass filtered at 7 Hz to create a linear envelope^34^. To isolate changes to the timing of muscle activity within each gait cycle, and because treadmill speed was held constant between conditions, we used numerical integration to compute the area under the EMG vs percent gait-cycle curve. Lower-extremity joint angles, moments, and powers were calculated in Visual 3-D (C-Motion, Gaithersburg, MD). For conducting the inverse dynamics analysis, the inertial properties of each personalized exoskeleton were calculated from computer-aided-design models in SolidWorks (Daussalt Systems, Waltham, MA) and appropriately added to the subject-specific link-segment models in Visual 3D. For each participant, joint mechanics data were divided into individual gait cycles and then averaged across gait cycles for each walking condition. Intervals when participants touched or held onto the treadmill guardrails were excluded from analysis.

Joint power, computed as the product of the joint moment and joint angular velocity, was defined as positive when the joint moment or exoskeleton torque acted in the same direction as the angular velocity, and negative when the joint or exoskeleton torque acted opposite to the angular velocity^35^. The average rate of positive and negative joint work (mechanical energy generation and absorption, respectively) was calculated by taking the time-integral of the positive or negative portions of each joint power vs gait-cycle curve^24^. For the exoskeleton conditions, biological knee joint moment and power, defined as the net contribution from all biological tissues (i.e. muscles, tendons, ligaments, and potentially bony deformities), was calculated by subtracting the exoskeleton’s contribution from the total (biological + exoskeleton) joint moment and power determined from inverse dynamics^36,37^.

### Statistical Analysis

The biomechanics data were segmented from heel-strike to toe-off (stance) and toe-off to heel-strike (swing). After normalizing, we partitioned the stance phase into early (first 50%) and late (final 50%) segments. The kinematic and kinetic outcomes were then averaged across the gait segments (early stance, late stance and swing), limbs, and participants.

To evaluate the biomechanical changes underlying improvement in posture from knee extension assistance, we used repeated-measures ANOVA to determine if significant differences in angle, biological moment, and biological power existed between the three walking conditions (without the exoskeleton, exoskeleton assist-off, and exoskeleton assist-on) for the hip, knee and ankle joints. One participant (P7) did not demonstrate postural improvements when walking with the exoskeleton (i.e. no improvement in crouch) and was therefore removed from the group analysis (n = 6). If a significant main effect was found, post-hoc comparisons between the three conditions were made using the Protected Fisher’s Least Significant Difference method. Pearson’s product-moment correlation analysis was used to test for relationships between knee kinematics and kinetics, muscle activity, physical exam characteristics, and training duration. Statistical significance was set at α < 0.05 for all tests.

## Results

During exoskeleton assist-on, the exoskeleton motors provided near-constant extensor torque during the early stance (0.19 ± 0.02 Nm/kg (mean ± sd)), late stance (0.17 ± 0.01 Nm/kg), and swing (0.07 ± 0.01 Nm/kg) phases across participants (Fig. 1d). During stance, this torque amounted to an average of 53% of the mean unassisted biological knee moment; the range across individuals was 30–99%. The exoskeleton generated a mean of 0.04, 0.03, 0.12 W/kg of mechanical energy generation and 0.01, 0.17, 0.03 W/kg of mechanical energy absorption during early stance, late stance, and swing, respectively (Fig. 1e). When exoskeleton assistance was turned off (but operating in “frictionless” mode), negligible reaction torque was measured (mean <0.01 Nm/kg).

**Figure 2:**
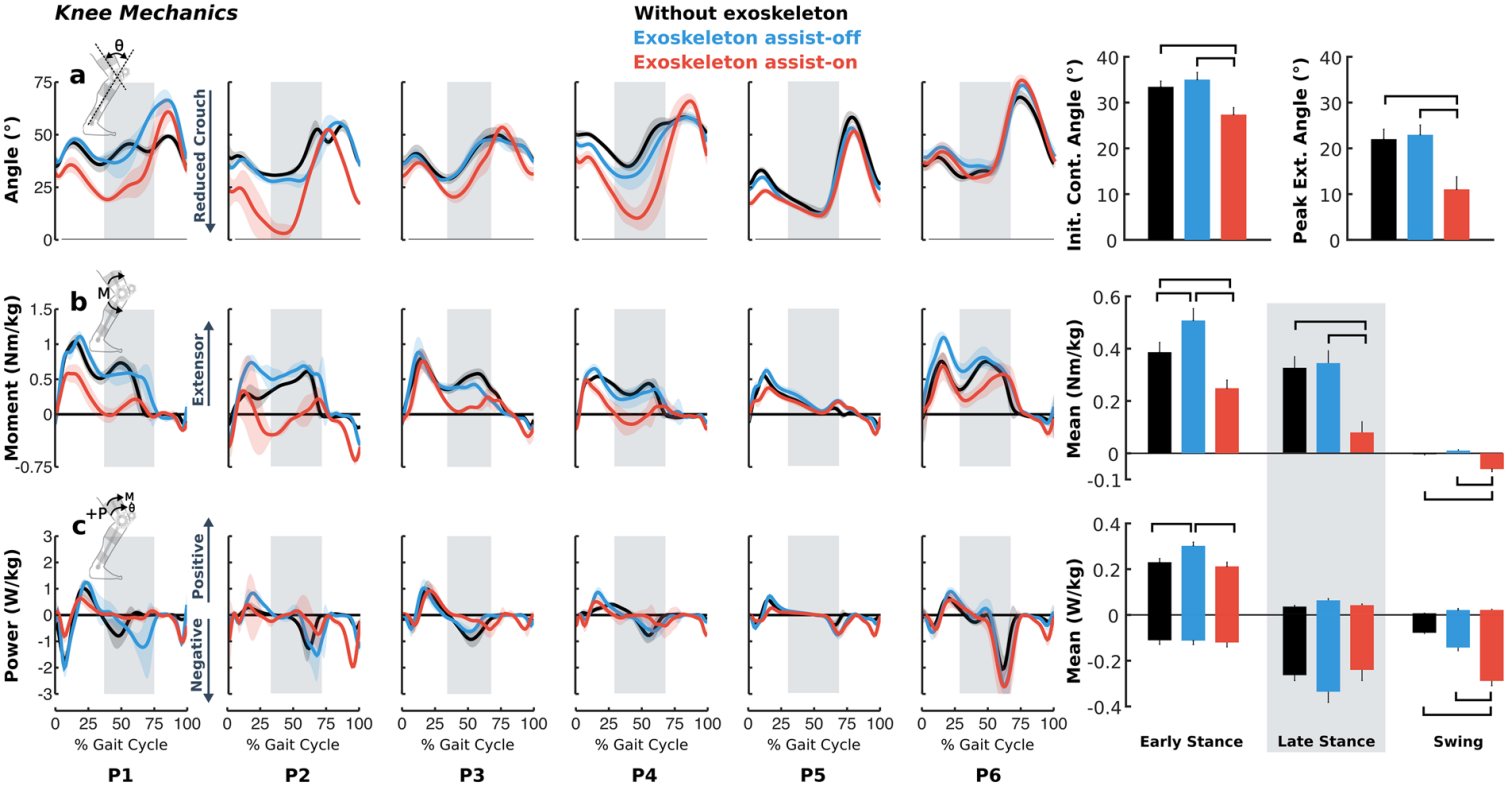
Biological knee joint angles (**a**), moments (**b**), and powers (**c**) during walking without the exoskeleton (black), and with exoskeleton assist-on (red) and assist-off (blue) plotted vs. percent gait cycle for each individual’s more affected limb. Line shading indicates ±1 standard deviation across gait cycles. Vertical gray shading indicates late stance phase. Bar plots on the right depict bilateral group-level differences (for all limbs) in knee angle at initial contact and peak knee extension (top) and bilateral group-level differences in biological knee moments (middle) and powers (bottom) averaged across gait phases. Error bars indicate standard error. Horizontal black bars indicate statistically significant differences between the corresponding conditions.

Walking with knee extension assistance affected stance phase kinematics and kinetics at the knee (Fig. 2). Compared to walking without the exoskeleton, walking with extension assist-on increased knee extension on average by 6° (p = 0.012) at initial contact while maximum knee extension during stance was increased by 12° (p = 0.001). There was no correlation between training duration and improvement in knee extension with the exoskeleton assistance (p = 0.67). The changes in posture were associated with altered biological knee moments. During early and late stance, the mean biological knee extension moment decreased by 0.14 Nm/kg (36%, p < 0.001) and 0.25 Nm/kg (76%, p < 0.001) respectively when walking with the exoskeleton assist-on. During swing phase the opposite effect was observed as biological knee moments increased by 0.06 Nm/kg (p = 0.005) in the flexor direction with the exoskeleton assist-on. This change coincided with an increase in mean knee joint energy absorption of 0.21 W/kg (269%, p < 0.001) during swing phase. Mean biological knee power during early and late stance phase was not statistically different between walking with the exoskeleton assist-on and walking without the exoskeleton. There was no group-level difference in knee angle when walking with the exoskeleton assist-off and walking without the exoskeleton; however, during early stance, the mean extensor moment increased by 0.12 Nm/kg (31%, p = 0.012) and knee joint energy generation increased by 0.07 W/kg (30%, p = 0.013) with the exoskeleton on compared to without the exoskeleton, an effect that was likely due to the increased mass of the device. Walking condition did not affect cadence (p = 0.17), step length (p = 0.34), or step width (p = 0.20) during treadmill walking at the same speed across conditions (Table 2).

**Table 2:** Exoskeleton, protocol, and spatiotemporal information.

Patient (ID)	Exo Mass (kg)	Walking Speed (ms^−1^)	Strides (#)			Cadence (Steps/min)			Step Length (m)			Step Width (m)		
			*Base*	*Off*	*On*	*Base*	*Off*	*On*	*Base*	*Off*	*On*	*Base*	*Off*	*On*
P1	3.1	0.40	15	7	13	119	109	111	0.17	0.20	0.22	0.28	0.27	0.25
P2	3.6	0.50	20	15	3	117	117	110	0.26	0.24	0.26	0.25	0.29	0.28
P3	4.5	0.45	32	12	8	122	120	105	0.21	0.28	0.26	0.17	0.26	0.20
P4	2.6	0.40	27	38	32	105	98	100	0.21	0.27	0.27	0.41	0.40	0.42
P5	3.8	0.55	31	31	30	89	106	95	0.21	0.22	0.22	0.23	0.23	0.21
P6	3.0	0.70	29	27	19	105	117	109	0.33	0.28	0.31	0.30	0.34	0.32
P7	3.7	0.45	4	6	12	108	118	110	0.37	0.34	0.36	0.21	0.21	0.20

Exoskeleton (Exo) mass is the combined total for both limbs. Treadmill walking speed remained constant across conditions. Strides: the number of strides available for analysis based on clean force plate strikes during steady-state walking; “base” refers to baseline walking, while “off” and “on” refer to walking with exoskeleton assist-off and assist-on, respectively.

**Figure 3:**
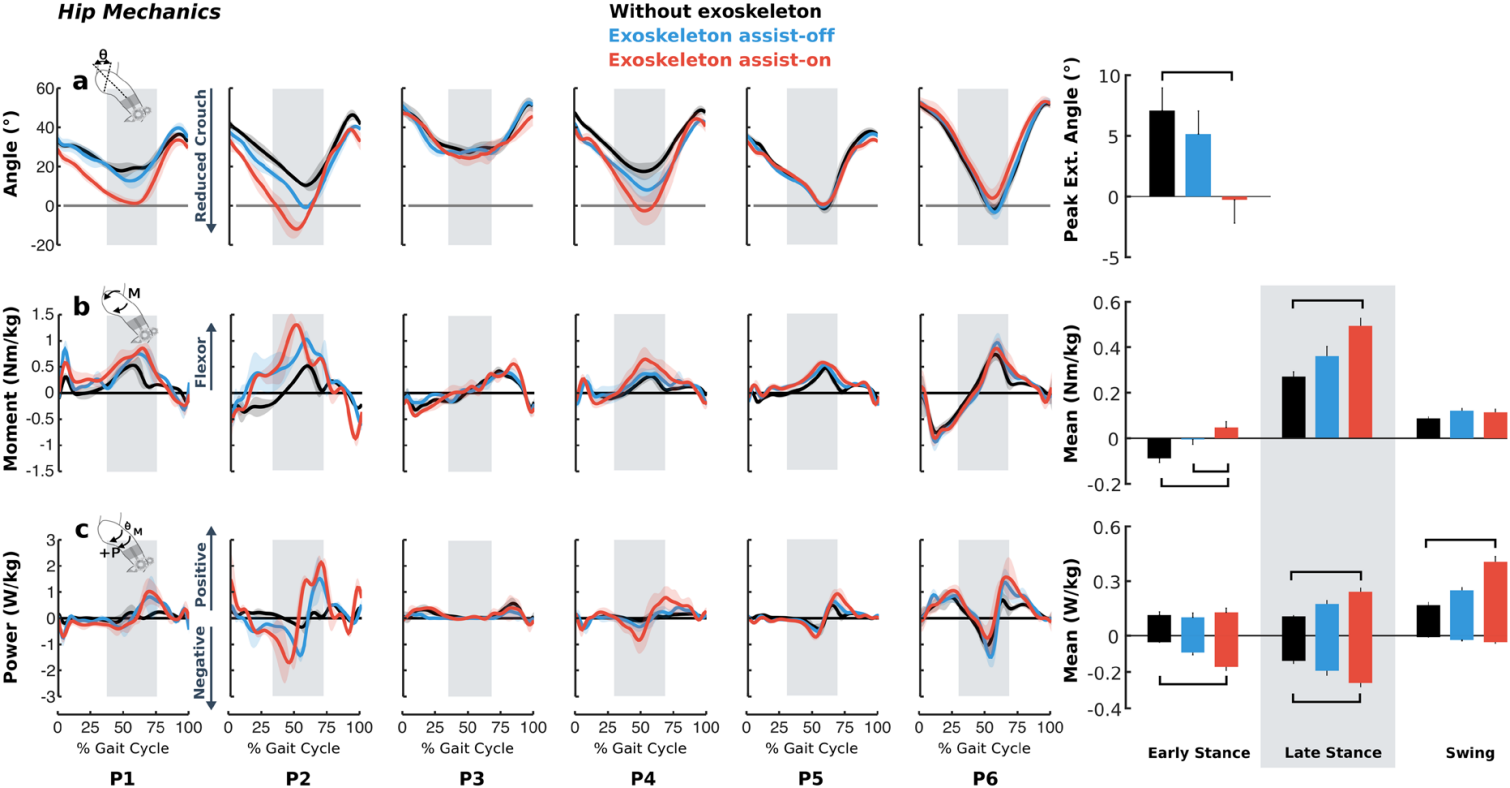
Biological hip joint angles (**a**), moments (**b**), and powers (**c**) during walking without the exoskeleton (black), and with exoskeleton assist-on (red) and assist-off (blue) plotted vs. percent gait cycle for each individual’s more affected limb. Line shading indicates ±1 standard deviation across gait cycles. Vertical gray shading indicates late stance phase. Bar plots on the right depict bilateral group-level differences (for all limbs) in the peak hip extension angle (top), biological hip moments (middle), and powers (bottom) averaged across gait phases. Error bars indicate standard error. Horizontal black bars indicate statistically significant differences between the corresponding conditions.

**Figure 4:**
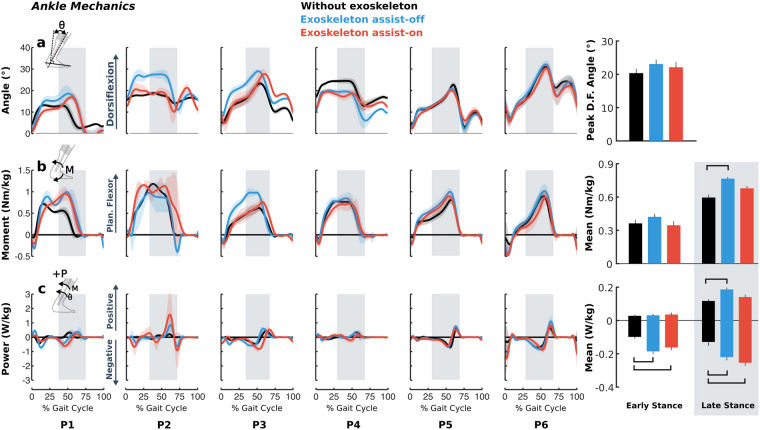
Biological ankle joint angles (**a**), moments (**b**), and powers (**c**) during walking without the exoskeleton (black), and with exoskeleton assist-on (red) and assist-off (blue) plotted vs. percent gait cycle for each individual’s more affected limb. Line shading indicates ±1 standard deviation across gait cycles. Vertical gray shading indicates late stance phase. Bar plots on the right depict bilateral group-level differences (for all limbs) in peak dorsiflexion angle (top), biological ankle moments (middle), and powers (bottom) averaged across gait phases. Error bars indicate standard error. Horizontal black bars indicate statistically significant differences between the corresponding conditions. Ankle mechanics data were not analyzed during swing because of the minimal effect of the ankle on gait during this phase.

Exoskeleton knee extension assistance had a more pronounced effect on the hip (Fig. 3) than the ankle (Fig. 4). Mid-stance hip extension increased by 8° (p = 0.001) with exoskeleton assist-on compared to baseline walking with an accompanying shift in mean biological hip moment toward the flexor direction by 0.13 Nm/kg (123%, p = 0.004) during early stance and 0.22 Nm/kg (86%, p = 0.001) during late stance. The change in limb posture was accompanied by increased hip joint energy absorption by 0.15 W/kg (377%, p = 0.001) and 0.12 W/kg (84%, p = 0.029) during early and late stance, respectively. Mean hip joint energy generation increased by 0.13 W/kg (133%, p = 0.005) during late stance, and by 0.24 W/kg (141%, p < 0.001) during swing. No significant differences were found at the group level for hip kinematics or kinetics between walking in the exoskeleton assist-off condition and without the exoskeleton.

No significant differences were seen in ankle angle between baseline and either exoskeleton assist-on or assist-off. Conversely, changes in biological ankle moments and powers were primarily driven by the presence of the exoskeleton itself, not the assistance as there were no significant differences between the two exoskeleton conditions (Fig. 4). Mean ankle plantar-flexor moment increased by 0.17 Nm/kg (18%, p = 0.009) during late stance for the exoskeleton assist-off condition compared to without the exoskeleton, while no significant differences from baseline were observed when the assist was turned on. Compared to walking without the exoskeleton, early stance energy absorption at the ankle increased by 0.09 W/kg (89%, p = 0.007) with assist-off and 0.06 W/kg (64%, p = 0.033) with assist-on, with no difference between these exoskeleton conditions. During late stance, energy absorption increased compared to baseline by 0.09 W/kg (70%, p = 0.005) and 0.13 W/kg (96%, p < 0.001) for assist-off and assist-on, respectively with no differences across exoskeleton conditions. Energy generation at the ankle increased by 0.07 W/kg (60%, p = 0.031) during late stance for exoskeleton assist-off compared to without the exoskeleton, while no significant difference was found between exoskeleton assist-on and the other conditions.

**Figure 5:**
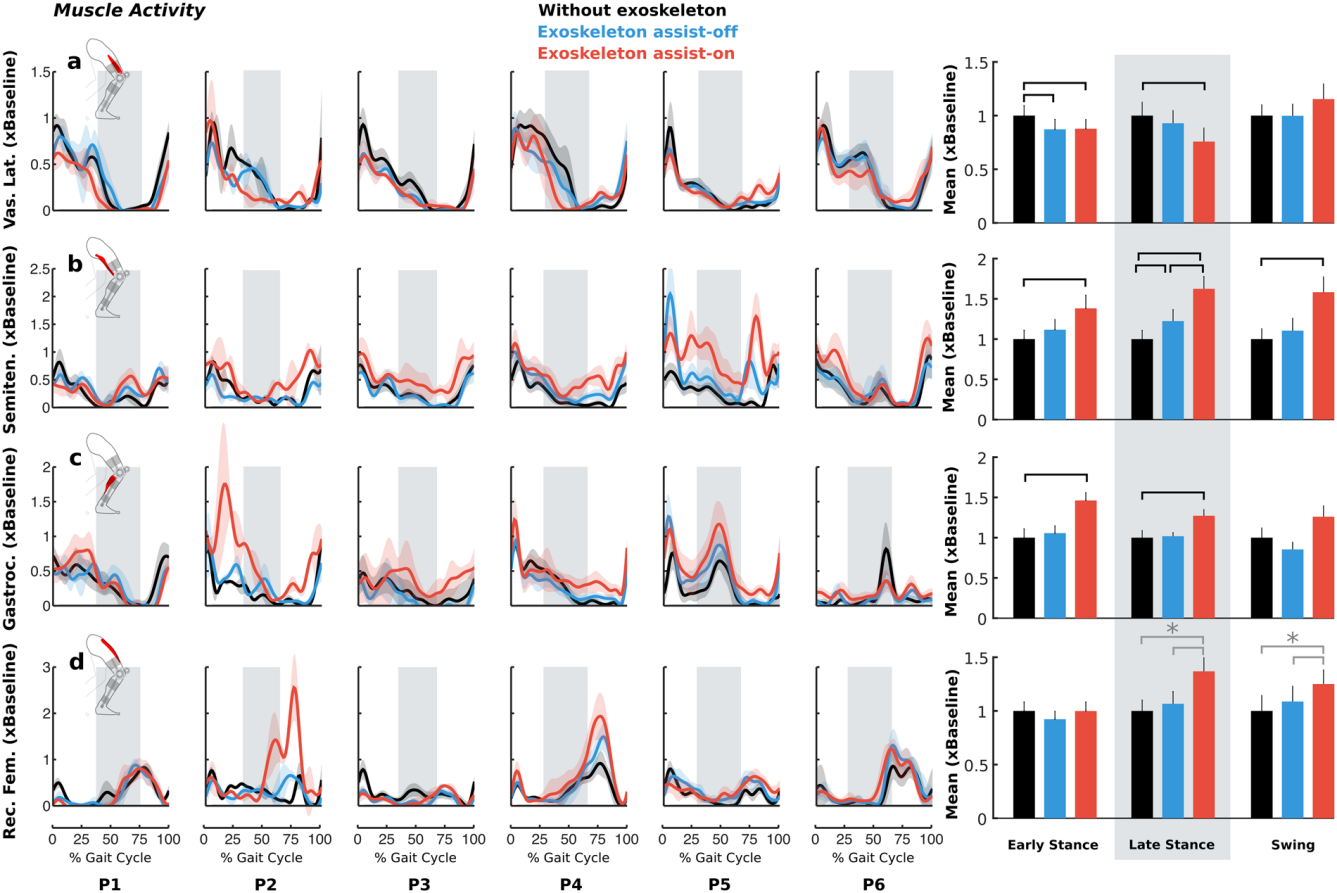
Muscle activity for the vastus lateralis (**a**), semitendinosus (**b**), gastrocnemius (**c**), and rectus femoris (**d**) during walking without the exoskeleton (black), and with exoskeleton assist-on (red) and assist-off (blue), normalized to the without exoskeleton (baseline) trials and plotted vs. percent gait cycle for each individual’s more affected limb. Line shading indicates ±1 standard deviation across gait cycles. Vertical gray shading indicates late stance phase. Bar plots on the right depict bilateral group-level differences (for all limbs) of muscle activity averaged across gait phases. Error bars indicate standard error. Horizontal black bars indicate statistically significant differences between the corresponding conditions. Asterisks and horizontal gray bars indicate that significant differences in rectus femoris activity were found for terminal stance/early swing (75–100% of the stance and 0–50% of swing) for the corresponding conditions.

Walking with exoskeleton assist-on altered stance phase muscle activity (Fig. 5). Compared to unassisted walking, semitendinosus activity increased by a mean of 38% (p = 0.019) during early stance, by 62% (p < 0.001) during late stance, and by 58% (p = 0.001) during swing for exoskeleton assist-on while the only difference in assist-off was observed in late stance phase when semitendinosus activity increased by 22% (p = 0.045). Semitendinosus activity for assist-on was 25% greater (p = 0.002) compared to the assist-off condition during this phase. Taken together, these results demonstrate a knee flexor response to the powered assistance. In early stance, vastus lateralis activity decreased by 12% (p = 0.046) and 13% (p = 0.038) for walking with exoskeleton assist-on and assist-off, respectively, compared to without the exoskeleton. Vastus lateralis activity during late stance decreased by 24% (p = 0.024) when walking with exoskeleton assist-on compared to without the exoskeleton, with no statistical differences between assist-off and baseline for that phase. The only differences in medial gastrocnemius activity from baseline were observed when walking with exoskeleton assist-on, as activity increased by 46% (p = 0.004) during early stance and by 27% (p = 0.007) during late stance compared to without the exoskeleton. No group-level statistical difference was found for the rectus femoris averaged across early and late stance, and swing. Since the rectus femoris functions across stance and swing phases, an *a posteriori* analysis found that in terminal stance/early swing (75–100% of the stance and 0–50% of swing) activity increased by 61% (p = 0.009) during walking with assist-on compared to walking without the exoskeleton and by 37% (p = 0.045) compared to exoskeleton assist-off. A comparison of peak muscle activity for each muscle is presented in Supplemental Fig. 1. During exoskeleton assist-on compared to without the exoskeleton, peak vastus lateralis activity decreased 19% during stance, peak semitendinosus activity increased 20% and 35% during stance and swing, respectively, peak gastrocnemius activity increased 28%, and peak rectus femoris activity increased 34%.

**Figure 6:**
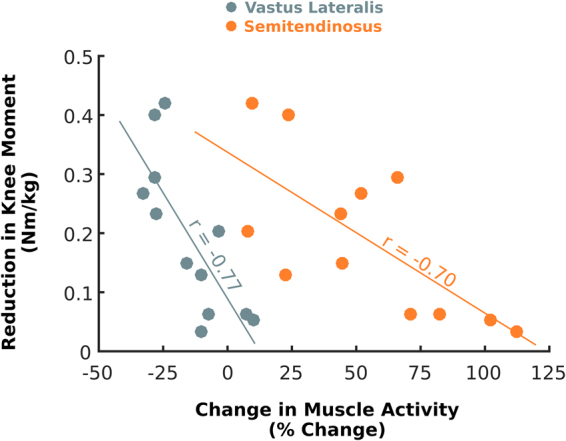
Mean stance-phase percent change in muscle activity plotted vs. mean stance-phase reduction in biological knee moment between exoskeleton assist-on and without the exoskeleton for the vastus lateralis (gray) and semitendinosus (orange) muscles. Pearson’s product-moment correlation coefficients (r) are noted above each best fit line.

Comparing exoskeleton assist-on to without the exoskeleton, we found significant negative relationships between the change in stance-phase biological knee moment and change in EMG activity for the vastus lateralis (r = −0.77, p = 0.003) and semitendinosus (r = −0.70, p = 0.011) muscles (Fig. 6). No significant relationships were found between the change in knee kinematics and muscle activity.

In contrast to all other participants, P7 did not have improved knee extension when using the exoskeleton (Supplemental Fig. 2); P7 exhibited a significant increase in knee flexion at initial contact (assist-off: 7°, p < 0.001; assist-on: 5°, <0.001) and peak knee flexion during stance (assist-off: 8°, p < 0.001; assist-on: 8°, <0.001) compared to baseline, with no differences in knee flexion between the exoskeleton conditions. One potential contributor to the increased knee flexion in the exoskeleton was the free ankle joint of the exoskeleton (compared to AFO at baseline), a difference that resulted in increased dorsiflexion across the gait cycle for both assist-off (13°, <0.001) and assist-on (14°, <0.001) conditions compared to baseline (Supplemental Fig. 3). While biological knee moments also increased in all three phases for both assist-off and assist-on compared to baseline, there was a significant reduction in biological knee moment in the assist-on compared to assist-off mode. In terms of power, the largest changes at the knee were in energy absorption, which was increased compared to baseline for both assist-off and assist-on modes in all three phases.

## Discussion

The primary aim of this study was to investigate how utilizing robotic assistance to increase knee extension in individuals with crouch gait from CP affects biomechanics of the entire limb, including joint angles, moments, and powers. Our findings support the hypothesis that providing a knee extensor torque during stance would result in an increase in knee extension coupled with a decrease in the biological knee extensor moment. The application of external extensor torque about the knee reduced the biological knee extensor moment across the entire gait cycle, with the largest reduction occurring during late stance. Changes in muscle activity largely corroborated our kinematic and kinetic findings, as reduced knee extensor moment was accompanied by reduced activity of vastus lateralis in stance while increased knee flexor moment and increased knee joint energy absorption were accompanied by increased semitendinosus activity during swing.

Favorable changes in knee dynamics exhibited by six of our seven participants in this study indicate the potential for robotic exoskeletons to treat crouch gait. Knee extension assistance increased lower-extremity extension by 12° at the knee and 8° at the hip during treadmill walking. This improvement in knee extension is similar to findings from invasive surgical interventions (10–20°)^5,6^ and floor-reaction AFOs (11°)^38^.

Our finding of similar biological knee power combined with reductions in both the knee extensor moment and excessive knee flexion suggest more efficient knee extensor muscle function with the exoskeleton. Providing a more upright posture and the accompanying favorable reduction in the knee extensor moment over extended periods of time could be critical for stopping or slowing the progression of crouch through adolescence and early adulthood^2^. During early stance, the knee joint exhibited a normal loading response despite the extension assistance from the exoskeleton. Biological knee joint power remained similar between walking with exoskeleton assist-on and without the exoskeleton. This was because the increased knee extension resulting from extension assistance was met with a concomitant reduction in the knee extensor moment.

In treating individuals with disorders like crouch gait from CP, which has multiple potential contributing factors, it is realistic that not all individuals would respond in the same way to robotic assistance. Not surprisingly, there was a clear variation in walking ability, both with and without exoskeleton assistance, across our cohort. Qualitatively, some individuals were able to walk more naturally in the exoskeleton assist-on condition than others. The additional challenge posed by walking on the treadmill may have also contributed to this spectrum of comfort. While P5 and P6 demonstrated clinically significant improvements in knee extension for at least one parameter (e.g. peak knee extension or knee extension at initial contact) for at least one of their limbs during the exoskeleton assisted treadmill walking trials, they exhibited greater improvement during over-ground walking^32^. One participant (P7) visibly struggled with walking on the treadmill at baseline with only 4 strides available for analysis (Table 1). P7 also struggled to acclimate to the exoskeleton during treadmill and over-ground walking as she exhibited increased knee flexion in both the assist-on and assist-off conditions, a posture that she likely adopted to increase stability and comfort level. As a result, she also showed an increased biological knee extensor moment across the stance phase with the exoskeleton (Supplemental Fig. 1). It should be noted that even in this more crouched posture, the biological knee moment was reduced with the exoskeleton assist-on compared to assist-off. This participant had greatly increased dorsiflexion during walking in the exoskeleton compared to baseline, suggesting that combining motorized knee assistance with an ankle condition that more closely resembled her prescribed AFO during typical walking would have improved outcomes. In this study, we chose to use a free ankle for all participants to isolate the effects of robotic assistance at the knee. In the future, combining the exoskeleton with an AFO at the ankle may give improved results for individuals in whom ankle dorsiflexion also contributes to crouch. Furthermore, additional walking practice with the exoskeleton would likely also improve outcomes.

Few significant correlations were found between changes in knee biomechanics during exoskeleton assisted walking and range of motion, spasticity, and muscle strength. These results are not unexpected. All participants met inclusion criteria of less than 5° of flexion contracture, assuring that their passive ROM enabled the opportunity for a positive response to the exoskeleton. The lack of correlation between kinematic response to the exoskeleton and spasticity and volitional muscle strength may be surprising initially, however our cohort was composed of independent ambulators; this requirement combined with the relatively small size of our cohort may have contributed to this finding. Spasticity was inversely correlated with change in knee moment during stance, such that those with high spasticity had less change in extensor moment which is not surprising because a larger or faster elongation of the quadriceps in the presence of assistance could result in a larger antagonistic response by those with greater spasticity. The strongest correlations were found between EMG activation and knee moments which is not surprising since greater force generation requires more muscle activation. Changes in stance-phase biological knee moments during exoskeleton assist-on were negatively associated with changes in vastus lateralis and semitendinosus muscle activity, indicating the importance of the neuromuscular modulation of both agonist and antagonist muscle groups in reducing knee extensor moment.

We found that the total (biological + exoskeleton) stance-phase knee joint moment during walking with exoskeleton assistance remained similar in magnitude to the biological knee joint moment during unassisted walking. As such, there was a nearly one-to-one change in the biological knee joint moment (54% reduction) corresponding to the level of robotic assistance (53% of the baseline, unassisted knee moment). The reduction in knee extensor muscle activity was modest (16%) and resulted in an activation profile more similar to typical gait (Fig. 5a)^39^. Thus, knee extensor activity did not decrease in direct proportion to the reduction in biological knee extensor moment (54%), resulting in an increase in lower-extremity extension with the exoskeleton assist-on.

Our results are somewhat comparable to findings from a study where researchers used a passive spring-based knee exoskeleton to provide external stiffness to the knee during walking in healthy adults; they found that the combined moment (biological + exoskeleton) remained invariant across a range of device stiffness^36,40^. Unlike our results, but not surprisingly considering the passive nature of their device and their investigation in individuals with normal knee function, they reported no significant changes in joint kinematics at ankle, knee, or hip.

During swing, the biological knee moment increased in the flexor direction, which opposed the exoskeleton-applied extension moment. While there was an improvement in knee angle at initial contact (6°), this improvement was less than the improvement in posture observed during stance. If the biological knee moment did not increase in the flexor direction, the ability of the exoskeleton to extend the knee prior to initial contact would have been improved. The change in biological knee moment from essentially zero (without the exoskeleton and exoskeleton assist-off) to flexor (exoskeleton assist-on) is corroborated by changes in the recorded muscle activity; knee flexor (semitendinosus) activity during late swing increased during walking with exoskeleton-assist on while knee extensor activity was not significantly different resulting in a shift of the biological knee moment toward the flexor direction. The ability of the exoskeleton to extend the knee prior to initial contact would have been improved if this increase had not occurred. These findings may indicate a neurologically based resistance to robotic assistance applied during open-chain movements where a rapid change in joint motion may be destabilizing. The increase in flexor activity may also constitute a spastic response to the increased knee extension velocity during late swing. Since increasing knee extension at initial contact is critical for improving crouch gait, techniques to limit the observed increase in the flexor moment and flexor muscle activity during late swing should be investigated, such as muscle toxin injections, functional electrical stimulation, or advanced control algorithms.

In addition to the observed changes at the knee joint, walking with knee extension assistance substantially affected joint dynamics at the hip. To maintain the whole-body base of support and avoid falling forward, the increase in knee extension was coupled with an increase in extension at the hip. This was evidenced by the fact that the participants who exhibited greater improvements in knee extension also exhibited greater improvements in hip extension, and offers one possible explanation for the increase in activity for the hip extensor muscles (i.e. semitendinosus). The increased hip extension was combined with a reduction in the hip extensor moment (shift of the hip moment in the flexor direction), leading to an overall more favorable dynamic posture.

Together, the observed biomechanical changes explain the proximal shift in joint power across the lower extremity during walking with knee extension assistance. During early-mid stance, the increase in semitendinosus activity combined with increased hip extension suggests that the hamstrings acted concentrically to improve posture and aid forward progression of the center of mass. During late stance of typical gait patterns, the hip flexors provide a propulsive force during “pull-off” and energy flows from the knee to the hip via bi-articular muscles^41^. While there were no changes in biological knee joint energy generation or absorption during early and late stance, mechanical energy absorption from the exoskeleton during late stance (pre-swing) likely contributed to the increase in biological hip joint energy generation. This was corroborated by the increase in rectus femoris muscle activity in terminal stance during walking with assist on compared to baseline and assist off.

Walking with knee extension assistance resulted in minimal changes in ankle function. With the foot planted on the ground, the external torque from the exoskeleton acted to rotate the thigh away from the shank, rather than the shank from the thigh, as demonstrated by the lack of change in ankle plantar-flexion angle, which likely contributed to the lack of increased energy generation at the ankle for exoskeleton assist-on while the additional inertia from the exoskeleton likely resulted in elevated energy absorption and generation during late stance at the ankle in the exoskeleton assist-off condition. Potential explanations for the slight increase in gastrocnemius activity during walking with knee extension assistance include attempts to increase ankle stability and counter-act the exoskeleton’s reaction torque on the shank.

Several of our results have implications for the control and implementation of exoskeletons used for the treatment and rehabilitation of pathological gait patterns. We observed no statistically significant differences in the total (biological + exoskeleton) knee moment across conditions. Thus, providing a constant amount of extension assistance increased knee extension while ultimately keeping the total knee moment the same. Further improvements in posture may be possible by incorporation of more sophisticated control methods that may reduce the total knee moment. For example, real-time estimates of the knee joint moment based on kinematic-kinetic joint coupling during crouch gait could be implemented in robotic control strategies to adjust assistance as a percentage of the instantaneous joint moment^42^. On another note, while improvements in posture were evident up the kinematic chain from the knee (i.e. at the hip), down the chain improvements (i.e. at the ankle) were not observed. These results may therefore suggest that adding small amounts of synergistic powered actuation at the ankle may provide further benefit to individuals with crouch gait, although this hypothesis should be tested experimentally. Considering the relatively short training duration (5 sessions), it is possible that more extended use would lead to additional biomechanical adaptations across the entire lower-extremity.

There are limitations with certain aspects of this study. We report data collected during treadmill walking, yet there may be small differences in how individuals with CP walk on a treadmill compared to over-ground. We used a treadmill for two reasons. First, due to the severity of the gait impairments, obtaining multiple clean, individual limb force plate strikes for each walking condition proved largely unattainable. Second, since gait dynamics are affected by walking speed, controlling walking speed, as is possible with a treadmill, is a desirable study design aspect to more precisely isolate the effects of exoskeleton assistance. The exoskeleton, which can also be operated under battery power, was tethered to a power supply during this protocol to ensure uninterrupted operation. Operating the exoskeleton under battery power (22.2v LiPo) may affect biomechanical outcomes because of the added mass (0.75 kg). A limitation of the exoskeleton design was that the knee joint was constructed as a hinge, which may have resulted in unwanted energy dissipation via movement of the device relative to the user. The present investigation only analyzed sagittal plane kinematics and kinetics; effects in other planes may exist. It is also unknown how walking with exoskeleton assistance may affect the metabolic cost of transport considering the added mass of the device, although the exoskeleton did actively compensate for its inertia and friction when the assistance was off. Another limitation was that we had a small sample size, and, like many clinical populations, our participants encompassed a range of neuromuscular deficits and their biomechanical responses to assistance were variable. Due to the exploratory nature of our study, our *post-hoc* statistical tests (Fisher’s Least Significant Difference) did not account for multiple comparisons. Extending our findings to other gait disorders or patient populations should be done with caution. The duration that our participants spent walking with robotic assistance was likely too short, and was not intended, to result in lasting neuromuscular changes when not wearing the device. Lastly, minor simplifications were made when completing the inverse dynamics analysis, including assumptions that the anatomical and exoskeleton knee joints were aligned, and that the exoskeleton moved rigidly with the anatomical segments.

In conclusion, we investigated the effects of a robotic exoskeleton providing knee extensor assistance on lower limb joint mechanics in individuals with crouch gait from CP. During treadmill walking at fixed self-selected speeds, the exoskeleton significantly improved dynamic gait posture and reduced excessive stance-phase extensor moments at the knee. Our findings provide evidence that individuals with gait deficits adapt to external knee extension assistance such that the total knee joint moment remains the same, even with changes in knee kinematics. Knee extension assistance improved posture and function at the hip, but not at the ankle. The ability for knee extension assistance to augment underlying lower limb muscle function and improve knee mechanics during this relative short exploratory study suggests further longitudinal investigations are warranted. Future work should investigate the use of the exoskeleton during longer-term robotic gait rehabilitation especially during periods of rapid physical growth when children are at greater risk for worsening crouch or in combination with other interventions which reduce spasticity (e.g. botulinum toxin injections or selective dorsal rhizotomy) or increase muscle length (orthopaedic surgery).

## Electronic supplementary material

Supplemental Material
