# Supplementary material for: The Effects of Exoskeleton Assisted Knee Extension on Lower-Extremity Gait Kinematics, Kinetics, and Muscle Activity in Children with Cerebral Palsy
Source: Sci Rep. 2017 Oct 18;7:13512. doi: 10.1038/s41598-017-13554-2 (PMC5647342; doi:10.1038/s41598-017-13554-2)
Supplement: Supplementary file 1 — Supplemental Material [file 41598_2017_13554_MOESM1_ESM.pdf]

Supplemental Material

The Effects of Exoskeleton Assisted Knee Extension on Lower-Extremity Gait Kinematics, Kinetics, and Muscle Activity in Children with Cerebral Palsy

Zachary F. Lerner[1,2], Diane L. Damiano[1], and Thomas C. Bulea[1*]

[1]Functional and Applied Biomechanics Section, Rehabilitation Medicine Department, National Institutes of Health, Bethesda, MD, USA.
[2]Department of Mechanical Engineering, Northern Arizona University, Flagstaff, AZ, USA.

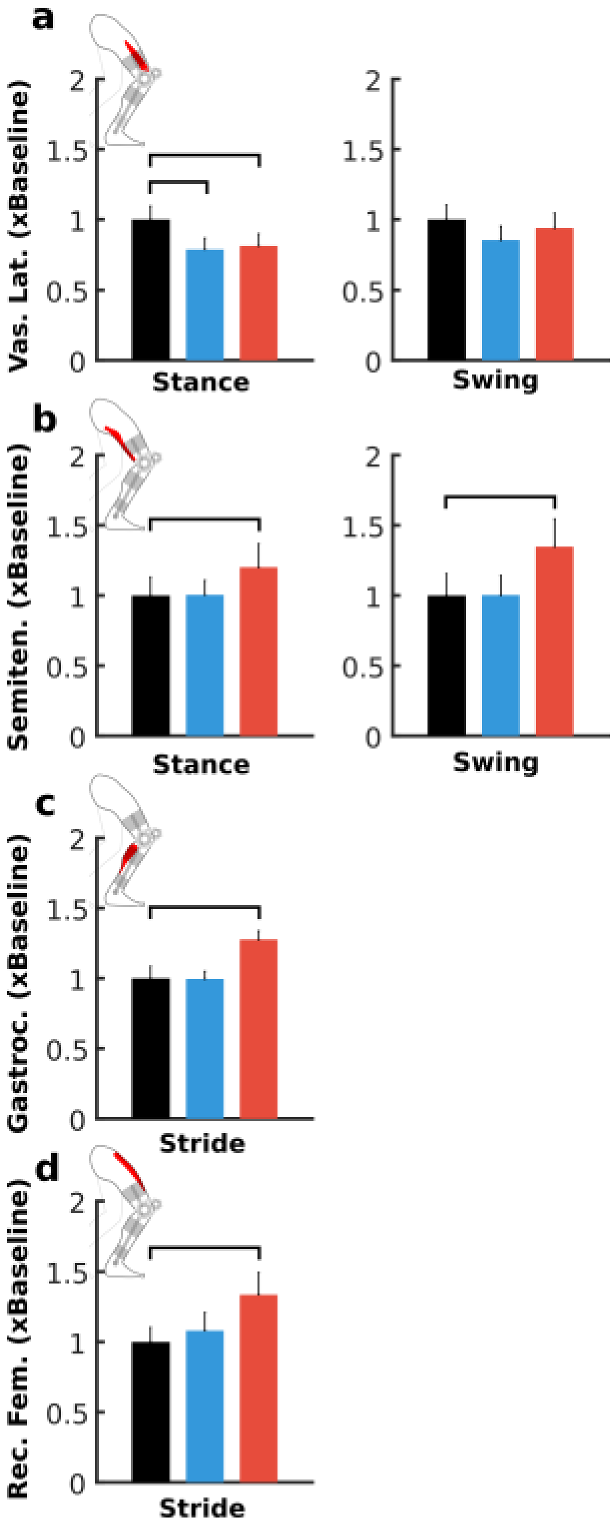

Supplemental Figure 1. Peak muscle activity for the vastus lateralis **(a)**, semitendinosus **(b)**, gastrocnemius **(c)**, and rectus femoris **(d)** during walking without the exoskeleton (black), and with exoskeleton assist-on (red) and assist-off (blue), normalized to the without exoskeleton (baseline) trials.

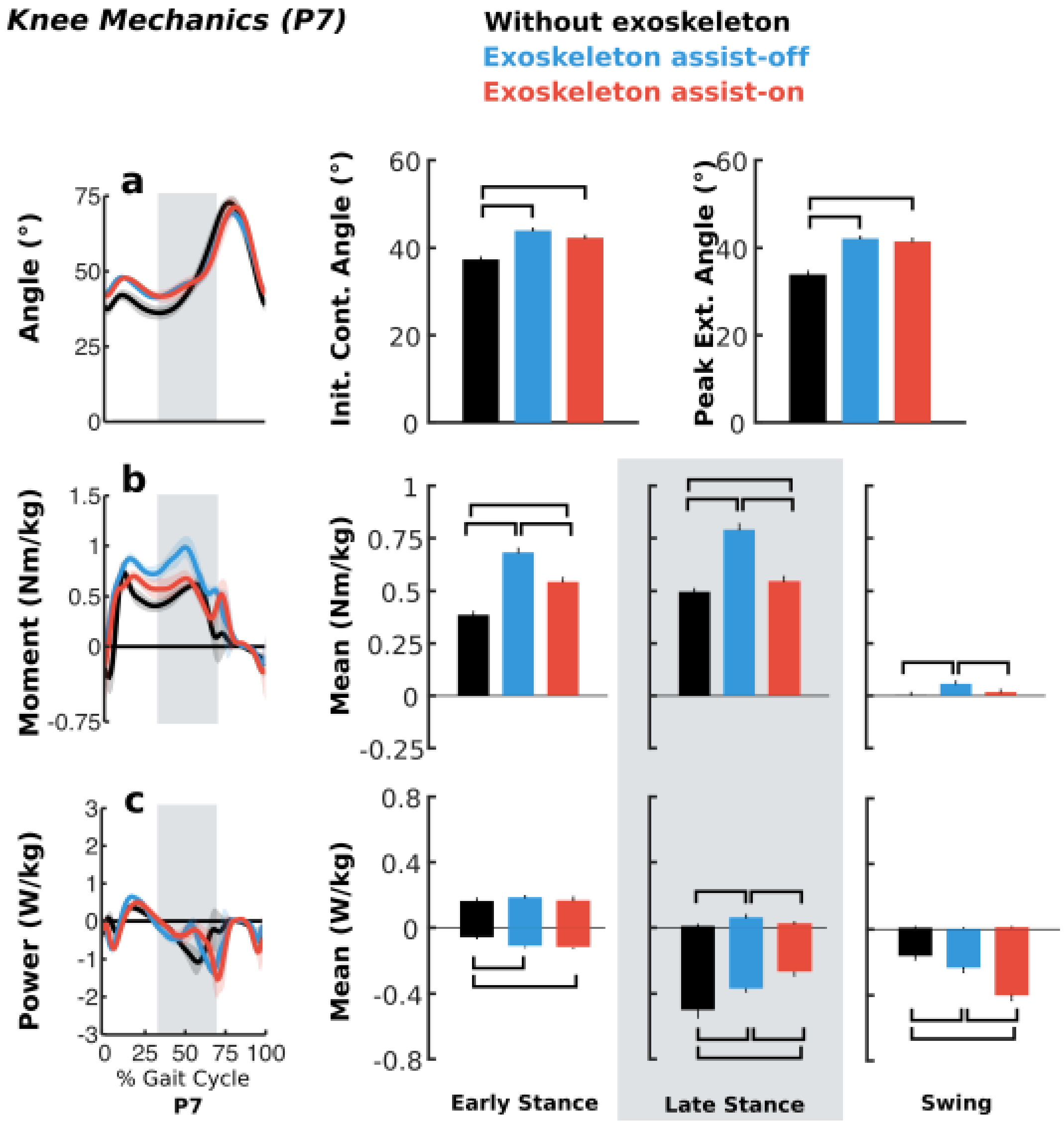

Supplemental Figure 2. Biological knee joint angles **(a)**, moments **(b)**, and powers **(c)** during walking without the exoskeleton (black), and with exoskeleton assist-on (red) and assist-off (blue) plotted vs percent gait cycle for the more affected limb of P7. Line shading indicates ± 1 standard deviation across gait cycles. The vertical gray shading indicates late stance phase. The bar plots on the right depict the bilateral average differences (for both limbs) of P7's knee angle at initial contact and peak knee extension (top) and average differences (for both limbs) of P7's biological knee moment (middle) and power (bottom) averaged across gait phases. Error bars indicate 1 standard deviation. The horizontal black bars indicate statistically significant differences between the corresponding conditions.

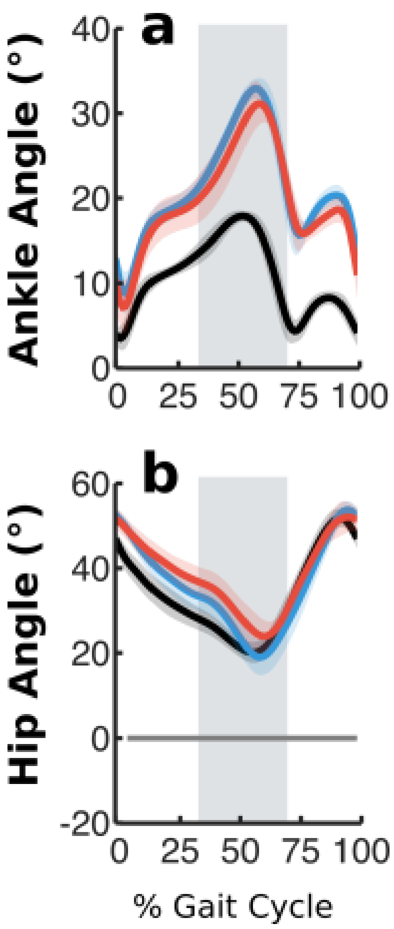

Supplemental Figure 3. Ankle (a) and hip (b) joint angles during walking without the exoskeleton (black), and with exoskeleton assist-on (red) and assist-off (blue) plotted vs percent gait cycle for the more affected limb of P7. Line shading indicates ± 1 standard deviation across gait cycles. The vertical gray shading indicates late stance phase.
